# Underuse, overuse, and guideline-based use of cervical cancer screening: social disparities in temporal screening trajectories in the French CONSTANCES cohort

**DOI:** 10.1186/s12905-025-03966-y

**Published:** 2025-10-15

**Authors:** Jeanne Sassenou, Virginie Ringa, Marie Zins, Anna Ozguler, Sylvain Paquet, Laurent Rigal

**Affiliations:** 1 Centre for Research in Epidemiology and Population Health (CESP), Hôpital Paul Brousse Bat 15/16 Av PV Couturier, 94807, Villejuif, France; 2 National Institute for Health and Medical Research (Inserm), Population-Based Epidemiologic Cohorts Units, UMS 011, Villejuif, France; 3 Université Paris-Saclay, 63 rue Gabriel Peri, Le Kremlin-Bicêtre, 94270 France

**Keywords:** Cancer screening, Cervical cancer, Socioeconomic disparities in health, Sequence analysis, Gynecology

## Abstract

**Background:**

The Pap test has been an important part of women's medical care for 40 years. Its utilization over time allows us to study both under- and over-screening. Our objective is to study the trends over time in each woman's screening status while simultaneously examining both its underuse and overuse.

**Methods:**

Our final sample included 55,141 women. We used sequence analysis methods to characterize trajectories of cervical cancer screening use for each woman. We then obtained 3 clusters of sequences. We performed bivariate analyses by comparing variables of interest according to each woman's cluster membership.

**Results:**

This study of Pap test reimbursement data shows that 70.7% of our sample was screened more often than necessary according to the guidelines for at least some periods of this study. The cluster analysis highlighted the consistency of the screening status. Once a woman "adopted" a screening rhythm, it appeared to continue over time. Most women who overused cervical cancer screening by Pap tests at the beginning of our observation period overused it throughout the follow-up period; the same consistency was found for those up-to-date at the start, and for those underscreened. The women in the overscreened group were in better health, younger, lived with a partner more often, and had the most favorable social characteristics.

**Conclusion:**

Our results showed an unequal distribution of social, demographic, and health characteristics across screening patterns. The majority of our sample was screened more often than necessary according to the guidelines then in effect. Once a woman "adopted" a screening rhythm, it appeared to continue over time.

## Introduction

Conducting regular cervical cancer screening to screen for cervical cancer is an effective way to prevent this disease [35]. Most professional societies across the world recommend its regular performance in women aged 25 to 65 years [4, 19, 21, 49]. Before 2020, the Pap test was largely an opportunistic screening test, that is, it depended on the initiative of individuals or their health-care advisor (although pilot programs tested organized screening before that date). Before 2020, French guidelines called for a cytological analysis to be performed every three years for women aged 25–65 after two normal Pap tests at ages 25 and 26 [27]. Until then, an HPV-test was performed only if the Pap test cytology results showed abnormal results: ASC-US (atypical squamous cells of undetermined significance), ACG (atypical glandular cells), ASC-H (atypical squamous cells that cannot exclude HSIL) or for colposcopy follow-up in the case of both LSIL (low-grade squamous intraepithelial lesion) and HSIL (high-grade squamous intraepithelial lesion) [29].

Numerous studies have looked at the underuse of Pap tests, or underscreening, during cross-sectional surveys that make it possible to obtain the woman's screening status at a given moment. The Pap test has been a part of women's medical care for 40 years. A woman can change her screening status at various points during her life — when a student, pregnant, while working, depending on her family situation, at retirement. Access to the databases covering reimbursement for health procedures allows us to understand the course of cervical cancer screening by Pap tests over time [18, 24]. It also enables the phenomenon of overscreening with Pap tests to be studied. Overscreening designates the performance of Pap tests too frequently or outside the recommended age range, without any justification. This phenomenon remains understudied despite its simultaneously personal and economic consequences. For individuals, it raises the risk of test-related adverse effects, such as pain or anxiety, and increases obstetric risks of preterm delivery among women who have had some cervical excision [37]. These superfluous examinations entail additional costs for both the individual and the health-care system [52].

Many studies have shown the existence of social inequalities related to underscreening [25, 26, 31, 39]. Other more specific social factors, such as migration history or overweight/obesity, both particularly interesting because of the discrimination they can cause [5, 9, 11, 28, 41], are associated with a higher prevalence of underscreening. Some factors related to underscreening have also been identified, although reversed or at least in different directions among overscreened women: the literature on this topic remains sparse [3, 6, 36].

Our objective is to study the trends over time in each woman's screening status while simultaneously examining both its underuse and overuse and to compare characteristics between statistical clusters for cervical cancer screening in a very large French cohort [17, 50].

## Method

### Study population

This study analyzes data from the CONSTANCES cohort, a general-purpose epidemiologic cohort designed to study a wide range of health problems in the general population. CONSTANCES collects data on personal, behavioral, occupational, and social factors from self-administered questionnaires at inclusion and afterwards, as well as from health examinations. Other information, such as reimbursement data (visits to doctors and other health professionals, reimbursement of the cost of care), is collected from databases of the SNDS (the national health data system) and the NHIF (French national health insurance fund). The SNDS is a health database built from reimbursement data from the French health insurance system. It make it possible to link health insurance data, hospital data, the medical causes of death, disability-related data, and a sample of data from supplementary health insurance organizations [30].

Inclusion in this cohort began in 2012 and concluded in 2021 with more than 220,000 cohort members (men and women) from the French population, aged 18 to 69 years [22]. Data considered here were collected at inclusion and come from women’s self-completed questionnaires about their socioeconomic, national, ethnic, and medical characteristics and linked SNDS/NHIF data about Pap test reimbursement. The National Data Protection Authority (authorization n◦910,486) approved the CONSTANCES cohort and its studies, which was then approved by the National Medical Council and the Institutional Review Board of the National Institute for Health and Medical Research (Institut National de la Santé et de la Recherche médicale). All participants provided written informed consent [15].

### Measures

These measures come from questionnaires completed for the CONSTANCES cohort by participating women. Immigrant status was constructed from three variables: the woman’s country of birth, her parents’ country of birth, and her administrative status (foreigner, French nationality born in France, naturalized). The variable was divided into three main categories: nonimmigrant women (born in France with both parents born in France), second-generation immigrant (born in France to at least one parent not born in France), and first-generation immigrant women (born abroad and living in France).

The standard divisions of BMI were considered: underweight or normal weight (BMI < 25 kg/m^2^), overweight (BMI [25–30]), and obesity (BMI ≥ 30). Age was used as a categorical variable in 5-year age groups."Living with a partner” was a binary variable. Financial difficulty was a 4-category variable, ranging from no financial difficulties to several years of financial difficulties. Monthly household income in euros was reported in five categories: < €1500, [€1500–2100[, [€2100–2800[, [€2800–4200[, and ≥ € 4200. Level of education was a 4-category variable based on success at the baccalaureate (school-leaving or “bac”) examination (some post-secondary education, completed high school and passed the bac exam, did not pass the bac exam, no secondary school diploma).

To quantify gynecologist visits we used the administrative record of health care consumption to determine if a woman been reimbursed for a gynecologist visit in the year before her inclusion. To characterize health status, we used women's perceived health status in four categories: “very good,” “good,” “bad,” and “very bad. The *Centre for Epidemiological Studies Depression Scale* (CES-D) is a 20-item self-administered scale that measures psychic health over the past week [42]. It is an indicator of psychological health [38].

### Analysis

The first step of the study was to identify the trajectories of cervical cancer screening use for each woman. We used sequence analysis methods to characterize these trajectories. These methods, based on a holistic approach to individual trajectories adapted to categorical data [1], aim to address two main questions (Abbott and Hrycak, 1990): What — if any — recurrent patterns of individual trajectories exist? What explanatory variables are linked to these different trajectories?

A sequence is a time-ordered succession of periods during which a subject occupies a given state. In our study, we defined a sequence as the succession of 23 periods, each four months long, from 2012 to 2019 for each woman in our sample, that is, seven and a half years of follow-up. Each period was characterized by a state corresponding to her Pap test status. To define this status we used all the reimbursement dates for Pap tests available from 2009 through 2019 in the reimbursement database, as well as the theoretical expected Pap test dates according to the guidelines in effect before 2019. The discrepancy between these theoretical expected dates and those observed for the reimbursement enabled us to define a status for each period: overscreened if there were two or more Pap tests in the previous 3 years; up to date, if there was only one, and underscreened if there was none.

Sequence analysis methods aim to compare all individual sequences with each other to establish a dissimilarity matrix between sequences to which clustering methods can be applied to obtain a typology of the sequences. The Optimal Matching analysis (OMA) applied in public health [1, 2, 45] establishes the dissimilarity between two sequences as the minimum cost of the virtual editing operations necessary for the sequence of one individual to become identical to that of another. We then applied a"partitioning around medoids (PAM)"clustering algorithm to the dissimilarity matrix computed by OMA to obtain clusters of individual sequences based on their similarity. The PAM algorithm is a partitioning algorithm based on k-medoids (class centers) that minimizes the total dissimilarity between individuals within each cluster [32]. Its limitations are that the number of clusters must be set in advance and that it depends on the choice of initial medoids. To circumvent the latter issue, the initial medoids are based on the class centers from a hierarchical ascending classification (HAC). To choose the optimal number of clusters, we tested partitions with 2 to 5 clusters, and we selected the partition containing the most relevant and homogenous clusters that were also sufficiently large (> 5% of the population).

We graphically represented the individual sequences for our entire sample and then distributed them into clusters. We also present the means of the screening statuses, distributed by period in each cluster.

To compare characteristics between clusters for cervical cancer screening, we performed bivariate analyses with chi2 tests by creating a variable of interest based on each woman's cluster membership.

We used R software, version 2023.06.2, with two specific packages: *TraminR* [20] and *WeightedCluste*r [51].

## Results

**Fig. 1:**
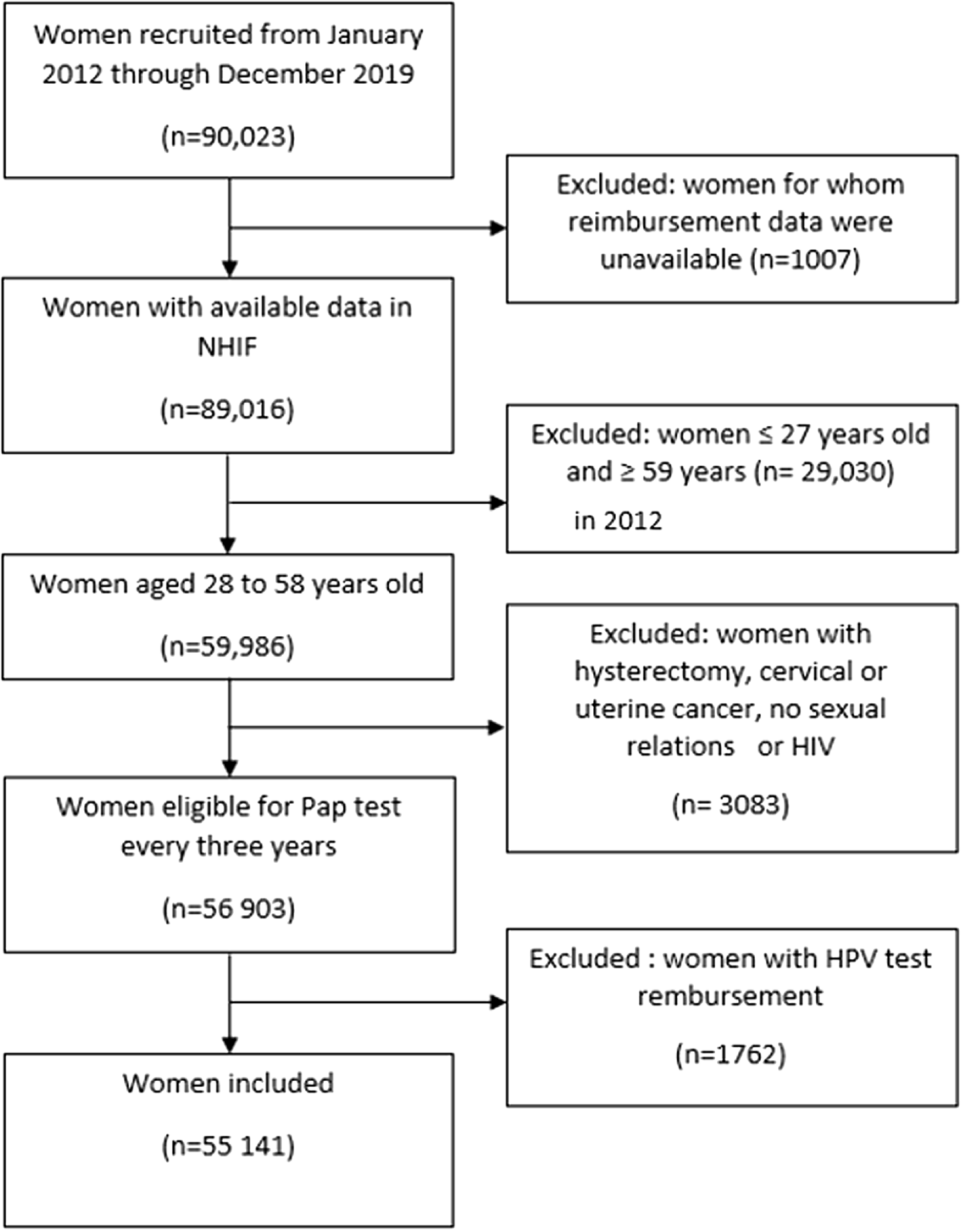
Flow chart

This analysis covers the 90,023 women recruited from January 2012 through December 2019 and their Pap test reimbursement data from January 2009 through August 2019. We excluded women whose NHIF reimbursement data were not available (*N* = 1007) and those ineligible for screening either at their inclusion or during their follow-up period (Fig. 1). This corresponded to women under the age of 27, as these women are required to undergo Pap test two consecutive years rather than every three years, and women over the age of 59 in 2012, as they would have become ineligible for follow-up. Because we aimed to quantify routine cervical cancer screening tests among asymptomatic women, we also excluded all women for whom an HPV test was reimbursed, because it was likely to have been associated an abnormal Pap test result, and thus not be normal screening (*N* = 1762). Our final sample included 55,141 women.

Most of our sample considered themselves in good or very good health, and most lived with a partner (Table 1) Slightly fewer than half the women had seen a gynecologist in the year before their inclusion. The majority of the women were not overweight and were not immigrants. Globally, our sample's socioeconomic characteristics were positive, with more than 80% reporting no current financial difficulties.

**Table 1:** Demographic, social and health characteristics of the population, *n* = 55,141

Characteristics	n (%)
**Age**	
[28–30]	458 (0.8)
[30–35]	5561 (10.1)
[35–40]	9643 (17.5)
[40–45]	10,832 (19.7)
[45–50]	8907 (16.2)
[50–55]	8149 (14.8)
[55–60]	8110 (14.7)
[60–65]	3407 (6.2)
**Health status**	
Very good	26,022 (49.1)
Good	21,533 (40.6)
Bad	5077 (9.6)
Very bad	354 (0.7)
**Lives with partner**	
No	13,013 (24.0)
**Migration history**	
Not immigrants	43,287 (80.0)
2nd-generation immigrants	6886 (12.7)
1st- generation immigrants	3935 (7.3)
**BMI (kg/m**^**2**^**)**	
< 25	34,926 (64.5)
[25–30]	12,673 (23.4)
> 30	6545 (12.1)
**Depression score (CESD)**	
Risk of depression	6856 (13.1)
**Saw a gynecologist in the year before inclusion**	25,608 (46.4)
**Financial difficulties**	
Never	32,581 (60.3)
In the past	14,527 (26.9)
In the past year	3452 (6.4)
For several years	3503 (6.5)
**Educational level**	
At least some postsecondary education	35,601 (65.6)
Completed high school. passed the “bac” (school-leaving exam)	8046 (14.8)
Did not pass the “bac” (school-leaving) exam	9237 (17.0)
No secondary school diploma	1357 (2.5)
**Monthly income (€)**	
> 4200	16,011 (31.1)
4200–2800	17,178 (33.3)
2800–2100	7946 (15.4)
2100–1500	5596 (10.9)
< 1500	4794 (9.3)

**Fig. 2:**
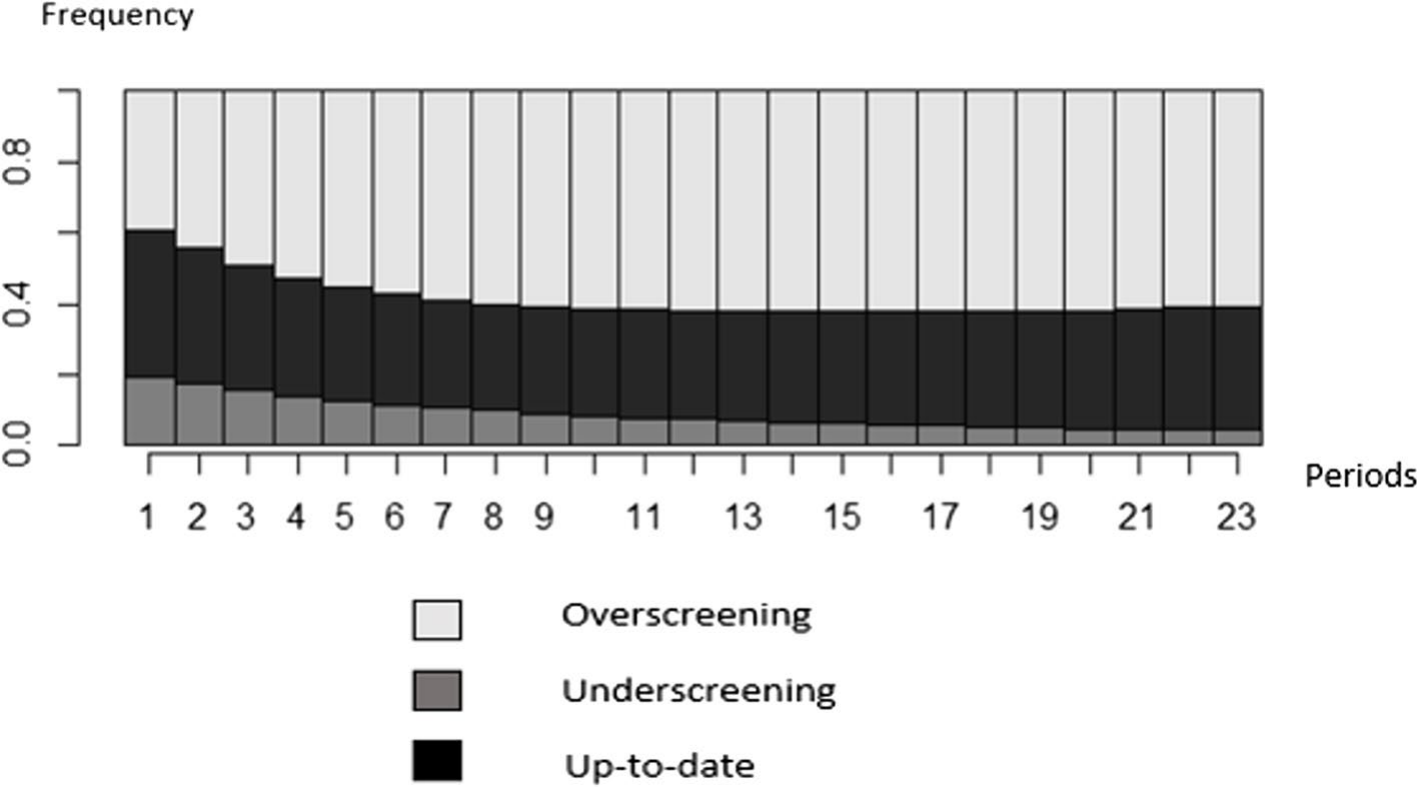
Graphic representations of Pap test screening status, cumulative in each period, *N* = 55,141. Reader's note: Fig. 2 presents the cumulative Pap test screening statuses in each period: during the first period, 20% of women were underscreened, 40% were up to date, and 40% overscreened

Among the 55,141 women in our sample and the 94 billion sequences possible, we observed only 1005 identical sequences. During the first observation period, from January through April 2012, 20% of the women in our sample underused screening, 40% were up to date, and 40% overused screening. The proportion of overscreened women increased over the course of the follow-up (Fig. 2).

**Fig. 3:**
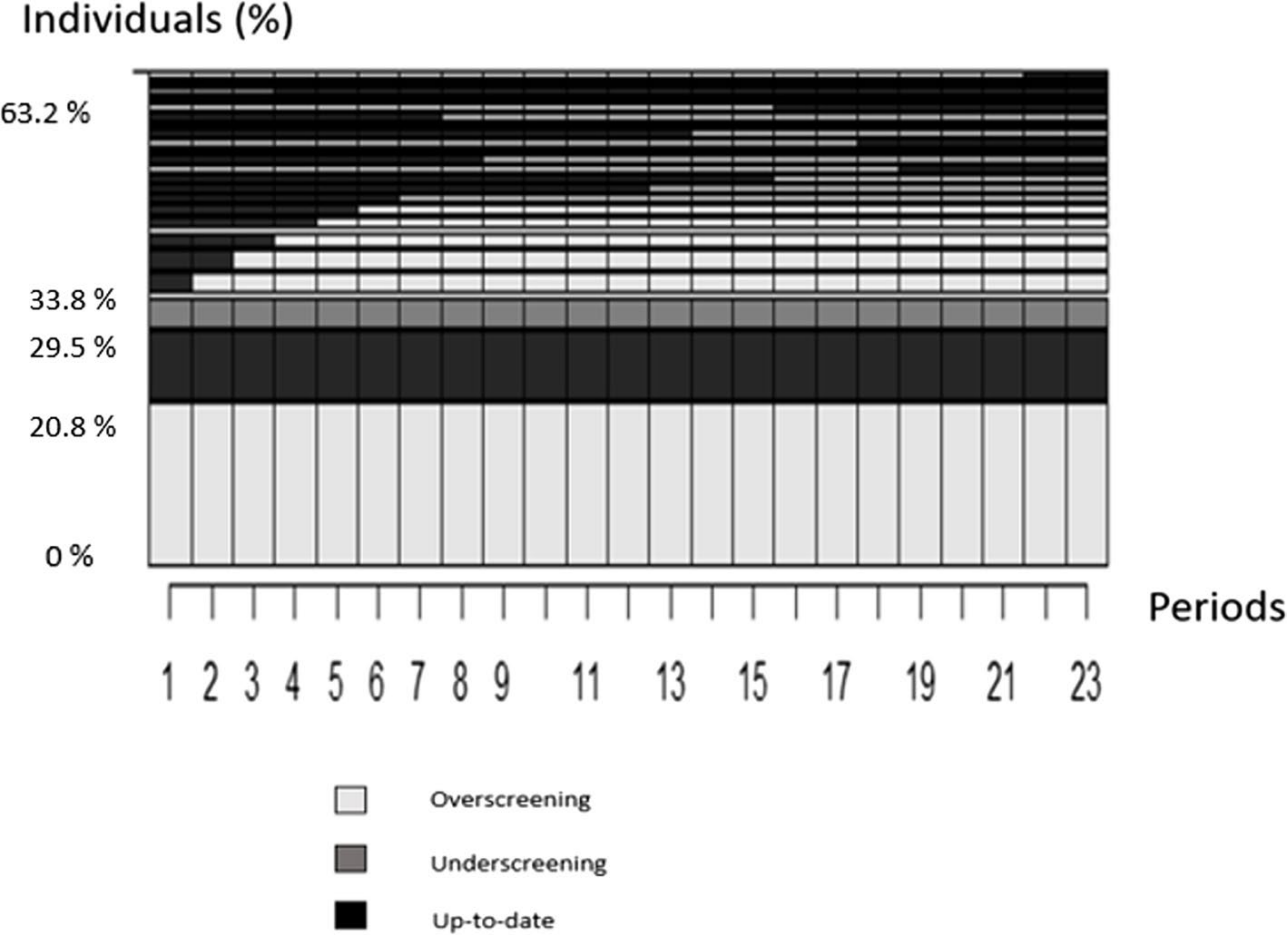
Graphic representations of the most frequent individual sequences of Pap test screening statuses, N=55,141 Reader's note: Fig. 3 presents the identical sequences and their stacking in decreasing order of cumulative frequency: the higher the sequence, the more women with this sequence. This figure represents the sequences of 63.2% of the women in our sample.

The majority had a consistent screening status throughout the entire 7.5 years of observation, regardless of whether they were overscreened, up to date, or underscreened (Fig. 3). The most frequent trajectory was that of women overscreened continuously throughout the 23 periods (20.8%), then 8.7% were up-to-date and 4.3% were underscreened throughout the follow-up. We observed sequences of women who were up to date at the start of the follow-up periods who then became consistently overscreened.

**Fig. 4:**
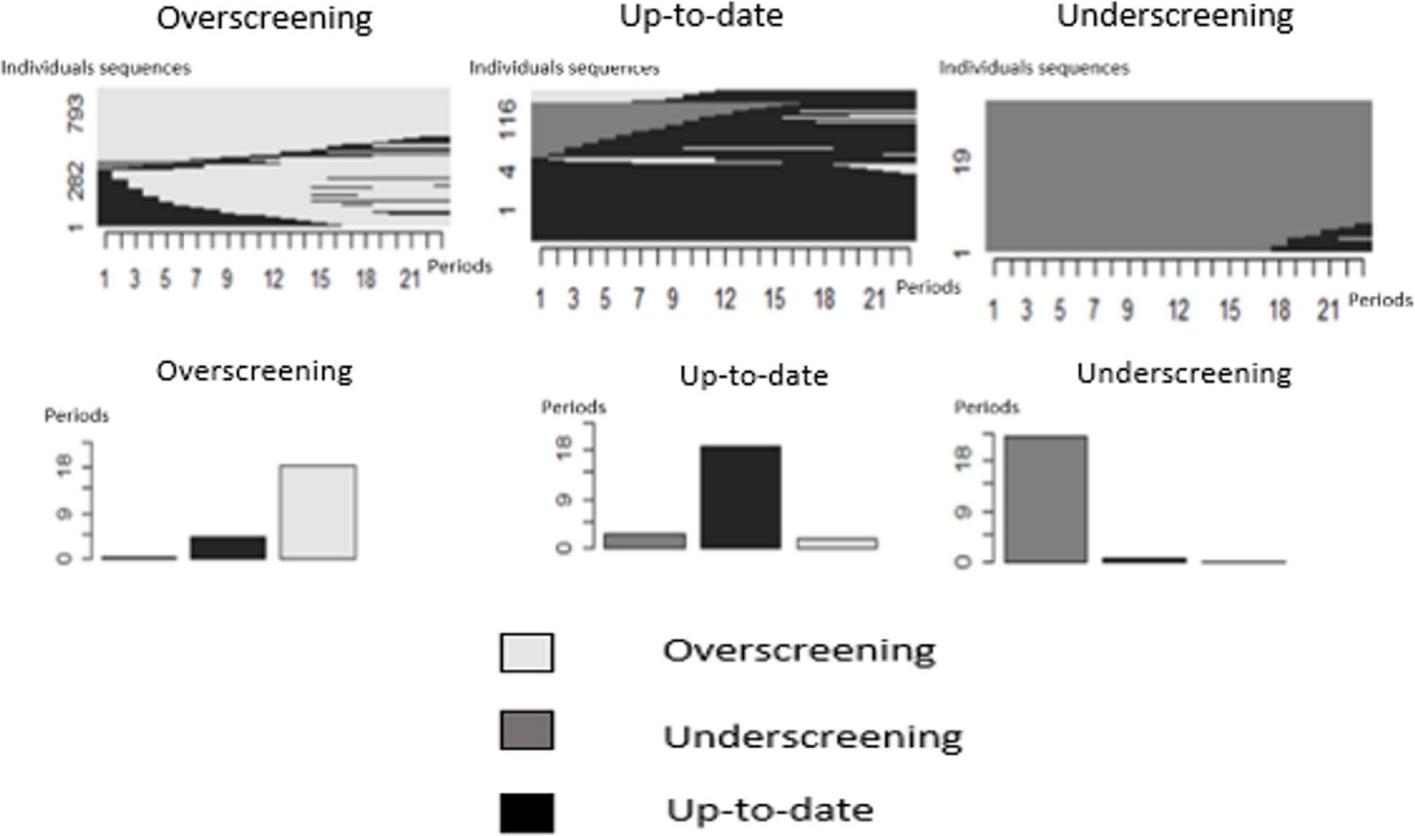
Graphic representations of the individual sequences and the means of the Pap test screening statuses composing the clusters, N = 55,141. Reader's note: Fig. 4 presents the individual sequences within each cluster according to their Pap test screening status classification. The identical individual sequences are regrouped: the higher the sequence, the more women it represents. The first cluster contained 793 different sequences, the second cluster 193, and the third 19. Diagrams present the mean time period for each status within three clusters

The distribution in three clusters allowed us to obtain a homogeneous and relevant distribution of our sample (Fig.4 ). The majority cluster contained 70.7% (*N* = 38,964) of our sample with 793 different sequences; the second most frequent cluster contained 23.8% (*N* = 13,126) of the women and 193 sequences, and the third cluster 5.5% (*N* = 3051) of the women and 19 distinct sequences. A majority of each cluster was composed of a single screening status: cluster 1 contained a majority of all overscreened statuses for the entire period, cluster 2 a majority of all up-to-date statuses, and cluster 3 a majority of all underscreened statuses.

We observed differences in women's profiles according to the cluster they belonged to. The women in the overscreened group were in better health, younger, most often lived with a partner, and had the most favorable social characteristics (Table 2). The women in the underscreened group most frequently reported financial difficulties, past or present. The proportion of women in the overscreened group had an educational level including some postsecondary schooling was 68.5%. Inversely, 26.8% of the women in the underscreening reported they had not passed the “bac” (school-leaving) exam. The women in the overscreened group more often had no family history of immigration history than the women in the up-to-date or underscreened groups Fig.

**Table 2:** Demographic, health, and social characteristics for each screening cluster, n = 55,141

	**Pap test screening clusters**			
	**Overscreened** **38,964** **(70.7%)**	**Up to date** **13,126** **(23.8%)**	**Underscreened** **3051** **(5.5%)**	***P* (Chi-2 test)**
**Age**				
[27–30]	0.8%	0.9%	0.9%	< 0.001
[30–35]	10.4%	9.6%	8.2%	
[35–40]	18.2%	16.7%	12.7%	
[40–45]	20.2%	19.5%	13.7%	
[45–50]	16.4%	16.0%	13.8%	
[50–55]	14.8%	14.7%	15.5%	
[55–60]	13.7%	15.7%	23.1%	
[60–65]	5.5%	6.9%	12.1%	
**Health status**				
Very good	51.0%	46.0%	38.4%	< 0.001
Good	40.0%	41.4%	45.0%	
Bad	8.4%	11.8%	15.2%	
Very bad	0.6%	0.8%	1.3%	
**Lives with a partner**				< 0.001
No	21.7%	27.1%	40.3%	
**Migration history**				
Not immigrants	81.5%	77.5%	72.1%	< 0.001
2nd-generation immigrants	12.4%	13.2%	15.1%	
1st-generation immigrants	6.2%	9.3%	12.7%	
**BMI**				
< 25	67.8%	58.0%	50.2%	
25–30	22.3%	25.9%	26.5%	
> 30	9.9%	16.1%	23.3%	
**Depression score (CESD)**				< 0.001
Risk of depression	12.0%	15.4%	17.6%	
**Saw a gynecologist in the year before inclusion**	55.4%	27.8%	11.4%	< 0.001
**Financial difficulties**				< 0.001
Never	63.1%	54.3%	49.5%	
In the past	25.7%	29.2%	31.7%	
In the past year	5.6%	8.0%	9.0%	
For several years	5.6%	8.5%	9.9%	
**Educational level**				
At least some postsecondary education	68.5%	60.3%	50.9%	< 0.001
Completed high school, passed the “bac” (school-leaving exam)	14.4%	15.7%	16.5%	
Did not pass the “bac” (school-leaving) exam	15.1%	20.7%	26.8%	
No secondary school diploma	2.0%	3.3%	5.8%	
**Monthly income (€)**				
> 4200	33.7%	26.1%	18.3%	< 0.001
4200–2800	34.5%	31.8%	25.7%	
2800–2100	14.6%	17.3%	18.3%	
2100–1500	9.9%	12.2%	17.6%	
< 1500	7.4%	12.6%	20.2%

## Discussion

This study of Pap test reimbursement data shows that 70.7% of our sample was screened more often than necessary according to the guidelines then in effect. Our study divides screening into 3 groups: a group of overscreened women who are socially advantaged, in good health and followed by a gynaecologist, a second group of underscreened women who are socially less advantaged, have less frequent gynaecological check-ups and are in poorer health, and an intermediate group up-to date. The cluster analysis highlighted the consistency of the screening status. Once a woman"adopted"a screening rhythm, it appeared to continue over time. Most women who overused cervical cancer screening by Pap tests at the beginning of our observation period overused it throughout the follow-up period; the same consistency was found for those up-to-date at the start, and for those underscreened. Our results were also consistent with a recent study investigating screening over time in another French cohort [18]. Starting screening is therefore a decisive stage: it determines what follows. This stability of screening makes analyses to identify and understand the differences within these three groups particularly useful. Our results confirm the findings of previous work about the factors related to underscreening and uncover those associated with overscreening. They also show an unequal distribution of social, demographic, and health characteristics, most favorable among overscreened women, and then among those up to date, and finally least advantaged among the underscreened.

One of the strengths of this study is that our longitudinal follow-up of Pap test reimbursement data enabled us to separate the overscreened women from those who were up to date. This is the first such study in France among women aged from 27 to 65 years. It highlights the predominance of overscreening and the existence of a continuum between overscreening and underscreening in relation to demographic, social, and health, characteristics.

CONSTANCES is the only French cohort to have included more than 200,000 volunteers from the general population. Two of the initial objectives in setting up this cohort were to study social inequalities in health and also specifically in women's health [15, 16]. The cohort studies combine data from several sources: self-administered questionnaires that explore numerous social characteristics, a specific questionnaire on women's health, and reimbursement data from the SNDS/NHIF), as well as medical data collected quadrennially during thorough health examinations.

The participation rate in the cohort is approximately 7% [47]. The women included in the cohort come from a more privileged background than the population as a whole because, despite stratification at the time of invitation, women from advantaged backgrounds ultimately participated at higher rates. This phenomenon is often observed in cohorts of volunteers [23]. For the 2018–2020 period in France, underscreening was estimated at 41% [46]. In CONSTANCES, underscreening at cohort inclusion was assessed at 21.3% [48], a rate corresponding to the prevalence of underscreening found in other French surveys [7] There are currently only a few studies on the prevalence of overscreening, and their results show marked variation [3]— undoubtedly reflecting the lack of standardization in measuring overscreening. Nonetheless, these studies all find socioeconomic inequalities in overscreening, with the most economically advantaged women most often overscreened. The lack of representativity of the study population may have resulted in underestimating underscreening and simultaneously overestimating overscreening.

All of the variables used except for those from the Pap test screenings come from the data collected at inclusion. These cross-sectional data do not take the changes in women's characteristics during follow-up into account.

An important limitation of our work is the lack of knowledge of the results of the Pap test. A positive result may lead to earlier cytological analysis in case of LSIL or HSIL one year after colposcopy [29]. In our study we excluded women who had had an HPV test because until 2019 it was only offered to women with a positive Pap result — 1762 women in our sample. In France in 2015 an estimated 3.9% of Pap tests were abnormal [29]. In our sample this would correspond to 2151 women. This allows us to estimate that ignorance of the Pap results resulted in overestimating overscreening for 0.8% of our sample.

The high prevalence of overscreening in our sample is striking. Besides its individual consequences, overscreening represents a substantial additional cost for the health care system [52]. In the USA [49], this additional cost has been estimated at 21.7 to 40 million dollars for young women who have a Pap test before the age of 21 years. Components of explanations can be sought simultaneously in the practices of the health care professionals who perform Pap tests and among the women who are screened.

The first guidelines for cervical cancer screening by Pap tests in France date from the beginning of the 1990 s [44]. The rhythm of screening recommended at that time was already a Pap test every three years from the age of 25 to 65 years. These guidelines were consistent with the European and international guidelines of this period [4, 43, 53]. This raises questions about health care professionals'lack of adherence to guidelines that have been stable for 30 years. Indeed any guidelines may conflict with the principles of professional independence and of the freedom to prescribe — both fundamental principles of medical practice in France [13, 14]. Guidelines are propositions intended to guide professionals in their decision-making, but if the latter consider that these recommendations are inappropriate for the medical situation or do not correspond to the latest scientific advances, they need not adhere to it. This change in"guideline-based"practices is more recent than those concerning screening. This discrepancy may explain why some health care professionals with more isolated practices do not follow these recommendations. In the USA, two studies analyzed the gap between Pap test practices and guideline adherence among a limited number of gynecologists. They reported that 33% of professionals, the professionals in, and 39% in the other, adhered to the guidelines about the frequency of screening [33, 54]. The principal reasons mentioned were their choice to provide better follow-up for their patients and to meet their expectations. Nonetheless, women's knowledge of the Pap test appears to be limited. In the USA, only 30% of the women questioned thought that the Pap test was simply a gynecologic examination involving the placement of a speculum [12]. A second study in France showed that 25% of the sample did not know whether it was necessary to perform a Pap test after HPV vaccination [10]. The frequency at which Pap tests should be performed is not well known, and the great majority of women think that the interval between two Pap tests is one year [10, 12].

Only a few years ago, gynecologists were the principal health care professionals performing Pap tests in France, far ahead of general practitioners, midwives, and clinical pathologists in laboratories that tested medical samples [34, 40]. They performed between 70 and 90% of Pap tests, depending on the region, and more than 70% of them charged fees for consultations that exceeded the fees on which health insurance reimbursements are based [8]. These fees above the amounts that are reimbursed is a factor that may well play a role in the social inequalities related to screening. In our work, more than half the women in the overscreened group had seen a gynecologist in the year before their inclusion, compared with only 11% for the women in the underscreened group. These results indicate that gynecologists play a role in overscreening.

## Conclusion

Our work shows the importance of the phenomenon of overscreening and its socially differentiated effect favoring the most advantaged women. These conclusions are elements that must be considered in seeking to improve screening coverage and to combat the social inequalities that it entails. The observation of women's consistency in the rhythm of screening opens up perspectives that can be applied to promote the importance of screening among women.

## Data Availability

The data is not freely available, but the analysis can be transmitted on request by the corresponding author. This site-https://www.constances.fr/- is a site about Constances cohort information.

## Appendix

Description women’s status in relation to cervical cancer screening

To be able to define women's status in relation to cervical cancer screening, we must define several indicators.

Let:

- ${PAP}_{n}$, be a Pap test performed at a date n
- ${t}_{1}$, be the date that the first PAP test was reimbursed from 01–01-2009
- ${t}_{n}$, be the date that ${PAP}_{n}$ was reimbursed
- ${f}_{n}$, be the theoretical date that ${PAP}_{n+1}$ was performed, that is, 1095 days after ${t}_{n}$
- ${t}_{F}$, be the date that the last Pap test available was reimbursed
- ${f}_{F}$, be the theoretical date that ${PAP}_{F+1}$ was performed
- ${d}_{n}$, be the interval between ${t}_{n}$ and ${t}_{n+1}$
- ${d{\prime}}_{n}$, be the interval between ${f}_{n}$ and ${t}_{n+2}$
- $d\_Start$, be the duration between ${t}_{1}$ and Jan 1, 2012
- $d\_Fin$, be the duration between ${t}_{F}$ and Dec 31, 2019
- ${S\_t}_{n}$, be the status at ${t}_{n}$
- ${S\_f}_{n}$, be the status on ${f}_{n}$
- ${S\_t}_{\text{01.01.2012}}$, be the status on 01–01-2012
- ${S\_f}_{F}$, be the status on ${f}_{F}$,

For all figures, the x-axis corresponds to the time.

General case

If ${d}_{n}<1095$ and ${d{\prime}}_{n}<0,$ then ${S\_t}_{n}=up-to-date$,${S\_t}_{n+1}=overscreened$ and ${S\_f}_{n}=overscreened.$

If the interval between two consecutive Pap tests was less than 1095 days and the date of performance of ${PAP}_{n+2}$ was before the theoretical date of the performance of ${PAP}_{n+1},$ we infer that the participant's status was up-to-date between the first two Pap tests, because she had an overscreened status until the theoretical date of ${PAP}_{n+2}$.

If ${d}_{n}<1095$ and ${d{\prime}}_{n}>0,$ then ${S\_t}_{n}=up-to-date$, ${S\_t}_{n+1}=overscreened,$ and ${S\_f}_{n}=up-to-date$.

If the interval between two consecutive Pap tests was less than 1095 days and the date of performance of ${PAP}_{n+2}$ was after the theoretical date of performance of ${PAP}_{n+1},$ we infer that the participant's status was up-to-date between the first two Pap tests, because she had a overscreened status until the theoretical date of ${PAP}_{n+1}$ and again became up-to-date until the date of ${PAP}_{n+2}$.

If ${d}_{n}>1095$ then ${S\_t}_{n}=up-to-date$, ${S\_f}_{n}=underscreened$, and ${S\_t}_{n+1}=up-to-date.$

If the interval between two consecutive Pap tests was greater than 1095 days, we infer that the woman had a status of up-to-date from the first Pap test until the theoretical date of ${PAP}_{n+1}$ because she had an underscreened status until the date of ${PAP}_{n+1}$.

If $${d}_{n}=1095,$$ then $${S\_t}_{n}=up-to-date$$, $${and S\_f}_{n}={S\_t}_{n+1}=up-to-date.$$

If the interval between two consecutive Pap tests was greater than 1095 days, we infer that the woman had an up-to-date status between the two consecutive Pap tests.

Case on Jan 1, 2012, date that the Pap test status period began

If $$d\_Start>0$$, then $${S\_t}_{\text{01.01.2012}}=underscreened.$$

If the first Pap test was performed after Jan 1, 2012, the participant would have an underscreened status from Jan 1, 2012, until her first Pap test, and then she would be up-to-date.

If $${d}_{Start}<0,$$ then $${S\_t}_{\text{01.01.2012}}=up-to-date.$$

If the first Pap test was performed before Jan 1, 2012, the patient's status would be up-to-date on Jan 1, 2012.

If ${d}_{Start}<0$ and ${t}_{n}<\text{01.01.2012},$ then ${S\_t}_{\text{01.01.2012}}=overscreened.$

If the patient had at least two Pap tests performed between Jan 1, 2009 and Jan 1, 2012, her status on Jan 1, 2012, was overscreened.

Case on Dec 31, 2019, study end date for the last Pap-test status period

If $${d}_{Fin}>1095,$$ then $${S\_f}_{F}=underscreened.$$

If the interval between the date of the last Pap test and Dec 31, 2019, was greater than 1095 days, the participant had an underscreened status on Dec 31, 2019.

If $${d}_{Fin}<1095,$$ then $${S\_f}_{F}=later than our colllection date.$$

If the interval between the date of the last Pap test and Dec 31, 2019, was less than 1095 days, the participant had an overscreened status on Dec 31, 2019.

If $${d}_{Fin}=1095,$$ then $${S\_f}_{F}=up-to-date.$$

If the interval between the date of the last Pap test and Dec 31, 2019, was equal to 1095 days, the participant had an underscreened status on Dec 31, 2019.
